# Lessons from digital technology-enabled health interventions implemented during the coronavirus pandemic to improve maternal and birth outcomes: a global scoping review

**DOI:** 10.1186/s12884-023-05454-3

**Published:** 2023-03-20

**Authors:** Imelda K. Moise, Nicole Ivanova, Cyril Wilson, Sigmond Wilson, Hikabasa Halwindi, Vera M. Spika

**Affiliations:** 1 Department of Geography & Sustainable Development, College of Arts and Sciences, University of Miami, 1300 Campo Sano Ave, Coral Gables, FL 33124 USA; 2 Global Health Studies Program, College of Arts and Sciences, University of Miami, 1252 Memorial Drive, Coral Gables, FL 33146 USA; 3 Department of Geography & Anthropology, University of Wisconsin-Eau Claire, Eau Claire, WI 54702-4004 USA; 4 Department of History & Political Science, Rogers State University, 1701 W. Will Rogers Blvd, Claremore, OK 74017 USA; 5 Department of Community and Family Medicine, School of Public Health, University of Zambia, P.O Box 50110, Lusaka, Zambia; 6 University of Miami, 1300 Memorial Drive, Coral Gables, FL 33124 USA

**Keywords:** mHealth, App, Mobile health technology, Telehealth, Telemedicine, COVID-19, Pandemic, Women’s health, Pregnancy, Prenatal care

## Abstract

**Background:**

Timely access to essential obstetric and gynecologic healthcare is an effective method for improving maternal and neonatal outcomes; however, the COVID-19 pandemic impacted pregnancy care globally. In this global scoping review, we select and investigate peer-reviewed empirical studies related to mHealth and telehealth implemented during the pandemic to support pregnancy care and to improve birth outcomes.

**Methods:**

We searched MEDLINE and PubMed, Scopus, CINAHL and Web of Science for this Review because they include peer-reviewed literature in the disciplines of behavioral sciences, medicine, clinical sciences, health-care systems, and psychology. Because our investigative searches reviewed that there is considerable ‘grey literature’ in this area; we did not restrict our review to any study design, methods, or place of publication. In this Review, peer-reviewed preprints were comparable to published peer-reviewed articles, with relevant articles screened accordingly.

**Results:**

The search identified 1851 peer reviewed articles, and after removal of duplicates, using inclusion and exclusion criteria, only 22 studies were eligible for inclusion in the review published from January 2020 to May 2022. mHealth interventions accounted for 72.7% (16 of 22 studies) and only 27.3% (6 of 22 studies) were telehealth studies. There were only 3 example studies that integrated digital technologies into healthcare systems and only 3 studies that developed and evaluated the feasibility of mobile apps. Experimental studies accounted 68.8% of mHealth studies and only 33.3% studies of telehealth studies. Key functionalities of the pregnancy apps and telehealth platforms focused on mental and physical wellness, health promotion, patient tracking, health education, and parenting support. Implemented interventions ranged from breastfeeding and selfcare to behavioral health. Facilitators of uptake included perceived benefits, user satisfaction and convenience. Mobile apps and short messaging services were the primary technologies employed in the implemented mHealth interventions.

**Conclusion:**

Although our Review emphasizes a lack of studies on mHealth interventions and data from pregnant women during the COVID-19 crisis, the review shows that implementation of digital health interventions during emergencies are inevitable given their potential for supporting pregnancy care. There is also a need for more randomized clinical trials and longitudinal studies to better understand the effectiveness and feasibility of implementing such interventions during disease outbreaks and emergencies.

**Supplementary Information:**

The online version contains supplementary material available at 10.1186/s12884-023-05454-3.

## Contributions to the literature

- Modalities and facilitators of uptake of mHealth and telehealth interventions implemented during the coronavirus pandemic is investigated as it impacted on obstetric and gynecologic care and outcomes.
- Key functionalities of the pregnancy apps and telehealth platforms are identified, and the focus of the implemented digital-enabled health interventions was on mental and physical wellness, health promotion, patient tracking, health education, and parenting support.
- Our findings demonstrate that digital technologies such as mHealth and telehealth have potential for supporting pregnancy care during emergencies and are vital health system strengthening tools globally.

## Background

Timely access to key obstetric and gynecologic healthcare has been shown to be an effective method for improving maternal and neonatal outcomes [1–3]; however, over the past 3 years, the COVID-19 pandemic has had severe effects on pregnancy care globally [4–14]. Several reports in the US have linked COVID-19 and resultant “Stay-at-Home” orders to changes in health service seeking behaviors among pregnant women [15, 16], a finding supported by a systematic review (*n* = 56 studies) and meta-analysis (*n* = 21 studies) conducted by UK researchers of global changes in prenatal care conducted in 2021 [14]. Another study conducted at a tertiary hospital in India noted a decrease in institutional deliveries (by 45%), but an increase in high-risk pregnancies including intensive care unit hospitalizations due to changes in pregnant women’s health seeking behaviors [17]. This finding was also reported by a UK study where the number of women seeking prenatal and postnatal appointments decreased [18]. The pandemic has undoubtedly heightened the risk factors generally linked with poor mental health in pregnancy such as anxiety, depression, and posttraumatic symptoms stemming partly from coronavirus infection fears, poor quality prenatal care, and restrictions on societal behaviors [19–21].

A common thread across the studies is in the observed relationship between the augmented need for structural changes to current prenatal care models including timing and frequency of prenatal care to meet the needs of providers, pregnant women, and their babies in different contexts and settings [7, 22–29]. Addressing these needs is key for achieving the United Nations Sustainable Development Goal (SDG) global target of less than 70 maternal deaths per 100,000 live births [30, 31] (from the current level of 152 deaths per 100,000 live births in 2020) [32]. However, while studies have been carried out on the impacts of COVID-19 on pregnancy care, facilitators of uptake of interventions or facilitators by which current prenatal care delivery models have been modified due to the coronavirus impact (e.g., without and /or with a hybrid of in-person and virtual visits) has not been synthesized in the literature.

Digital technologies (e.g., mobile phone and tablet apps and telehealth) have become vital health system strengthening tools globally [33, 34] mostly for overcoming healthcare service delivery challenges [35–40], and their use in pregnancy care has been augmented by the coronavirus crisis. Mobile health technology (mHealth) is defined as a component of electronic health used for delivery of health services using information and communication technology [41–44]. Another digital tool is telemedicine, a component of telehealth, and a practice of medicine utilizing technology to deliver care at a distance (telemedicine signifies clinician-patient interactions and consultations that happen remotely via phone, video calls, text messaging, or other formats) [45, 46]. Systematic reviews and studies conducted in both the ‘global North’ [47, 48] and the ‘global South’ [8, 49–56] have associated patients’ timely access to health services and improvements in obstetric and gynecologic outcomes with digital health interventions [43, 55, 57–62] such as test result notification [63], patient management [64], and real-time access to patient information and communication at different points of care [65–67].

Numerous studies have already demonstrated how digital technologies can address healthcare challenges such as those placed by the coronavirus [68, 69] on pregnancy care and health-seeking behaviors of pregnant women during the pandemic [45, 70, 71]. Additionally, studies evaluating provider and patient experiences with digital-enabled consultations and appointments during the coronavirus crisis report high usage, patient and provider satisfaction [45, 72–77], and that both personal and organizational factors motivate implementation [78, 79]. To note, a recent scoping review that used Google searches to evaluate commonly used apps by pregnant women found over 57 unique apps. Of the final evaluated 29 apps, 18 did not have comprehensive information for every stage of pregnancy [80]. This underscores a need to synthesize information regarding the usefulness and benefits of the implemented apps during the coronavirus crisis to support pregnant women, and whether app differences varied by intervention. Furthermore, while some research has been carried out on pregnant women and COVID-19 [14], no single study exists which assessed a combination of digital-enabled technology (mobile phone and telehealth) implemented during the coronavirus crisis to support pregnancy care. Understanding how and why the digital-enabled technology health interventions were implemented and how they impacted pregnancy care (prenatal, pregnancy and postpartum periods) offer a potential opportunity to improve pregnancy care, reduce costs, resources, and time.

We undertook a global scoping review to identify peer-reviewed articles that used mobile phone and telehealth health interventions to improve pregnancy care and/or outcomes during the COVID-19 pandemic. To our knowledge, this is the first study to uncover context, digital interventions, uptake facilitators, locations, study designs and outcomes in the implemented interventions aimed at supporting pregnancy care during the COVID-19 pandemic.

## Methods

### Overview

We conducted a scoping review, a method of knowledge synthesis guided by Arksey and O′Malley’s framework for conducting scoping reviews [81]. To ensure reviewer compliance with best practice guidelines for the conduct of scoping reviews, the PRISMA (Preferred Reporting Items for Systematic reviews and Meta-Analyses extension for Scoping Reviews) checklist and flow diagram [82] was followed closely, with minimal disagreements because we established a priori inclusion and exclusion criteria in consultation with a research librarian.

### Data sources

We conducted preliminary searches on COVID-19, pregnant women, postpartum, pregnancy, mobile phone apps, and interventions as well as combining the keywords on Google scholar and Sematic scholar in May 2022. These searches facilitated the delineation of the review scope of this study, research questions, and established eligibility criteria. Subsequently, we selected MEDLINE and PubMed, Scopus, CINAHL and Web of Science for this Review because they include peer-reviewed literature in the disciplines of behavioral sciences, medicine, clinical sciences, health-care systems, and psychology. Appendix A presents the alternative used key search terms used in this study. Because our investigative searches reviewed that there is considerable ‘grey literature’ in this area; study design, and outcome indicators studied varied widely, we did not restrict our review to any study design, methods, or place of publication. Peer-reviewed preprints were comparable to published peer-reviewed articles, with relevant articles screened accordingly.

### Inclusion and exclusion criteria

The scoping review included studies that leveraged digital health interventions (e.g., mobile phone, telehealth, or video conferencing) implemented to enhance access to and/or linkage to pregnancy care services such as to support the three stages of pregnancy (perinatal, pregnancy or postpartum) and improve maternal and neonatal outcomes. Two independent reviewers [IKM and NI] searched databases and grey literature for references of identified peer reviewed studies published from January 2019 up to May 2022.

We excluded studies published before the COVID-19 pandemic, study protocols [83, 84], commentary [9, 40, 46, 85–87] or viewpoint [26], thesis [88], or if they were qualitative studies focused on perception of users, mHealth conceptual models [26, 89] not published in English language or did not report on COVID-19. We excluded review papers or studies focused on only mobile phone apps and/or pregnant women without a link to COVID-19 [55, 90, 91]. Also excluded were studies that did not focus on an intervention (e.g., mental health, education, test, vaccines, lifestyle, or mental health).

### Screening and data extraction

Studies retrieved from each database were imported into Endnote X8 reference management software [92]. These studies were then imported into Covidence, a systematic online review management program [93] which allowed for screening of eligible studies. The screening process was completed in two phases after duplicates were removed. After the two of five reviewers [including IMK, NI] screened all articles independently at each stage, the authors screened all articles based on relevance of information contained in the title and abstract, and then determined their inclusion for full text review. We then compared the individual screening results and resolved discrepancies by consensus via discussion among the five researchers. Only studies that met the inclusion criteria underwent a full text review.

Two reviewers abstracted data which was then validated by a third reviewer, with sections assigned based on reviewers’ expertise.

### Quality assessment

To evaluate the methodological quality of all primary research studies, we utilized the Joanna Briggs Institute Critical Appraisal Checklist for Prevalence Studies [94]. We used the checklist to assess the extent to which the selected studies report on the likelihood of bias in nine topics of study design, conduct, and analysis as used in previous studies [95], ranging from phase 0 (poor quality) to 2 (high quality), with studies receiving a total quality score (ranged from 0-poor quality to 18- higher quality). We excluded studies with a total score of < 13. Five researchers [IMK, NI, CW, SW, and SV] independently evaluated each included study, and through discussion, we decided on any doubt about the quality of included studies.

### Data synthesis

We reviewed attributes of the included studies. Narratively synthesized were key implemented health interventions that supported the three stages of pregnancy. To categorize the selected studies, we adopted a conceptual model developed a priori based on existing literature on mHealth and maternal and child health [43]. Digital interventions were identified based on their purpose, modalities, facilitators for uptake (e.g., choices, perceptions), relevant actors (pregnant women, providers, mothers), study design, context or conditions required for program facilitators to activate or not, and mobile phone or telehealth intervention outcomes [43].

## Results

### Study characteristics

**Fig. 1:**
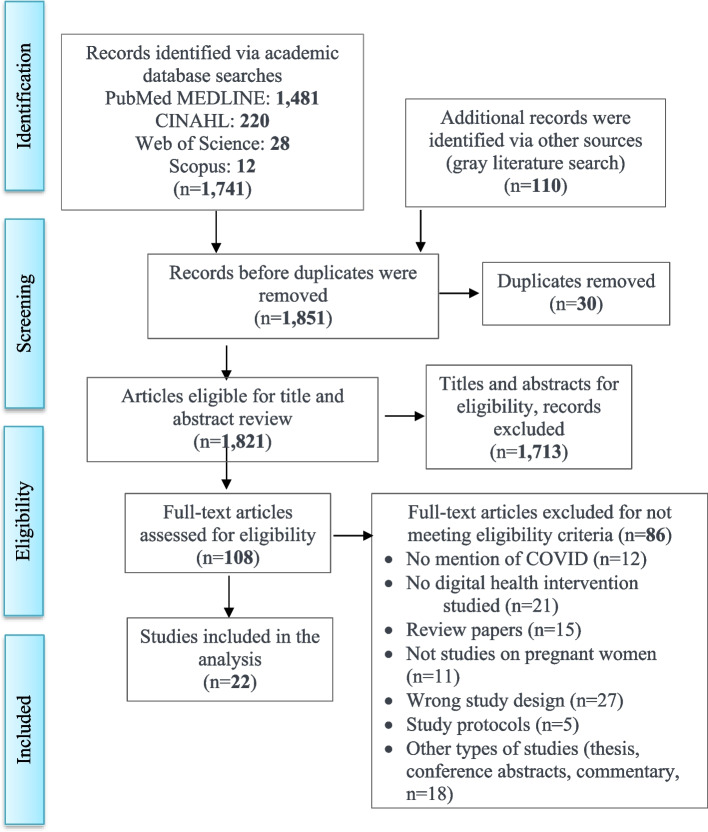
Prisma flowchart of information through the different phases of article extraction from the literature search

Figure 1 presents the PRISMA flow diagram used for the scoping review. The search yielded 1851 peer reviewed articles from select databases: PubMed MEDLINE (1481), CINAHL (220), Web of Science (28), Scopus (12) and grey searches (110). Of these, we excluded 30 duplicates. Of the remaining 1821 studies, 1713 studies were non-empirical, such as study protocols, thesis, commentary, viewpoints, or reviews. Based on title and abstract review, 108 studies were left for a full-text review, and of these, 85 studies were excluded (with 18 studies excluded after assessment for quality) leaving 22 studies (*n* = 16 mHealth studies and 6 telehealth studies).

**Fig. 2:**
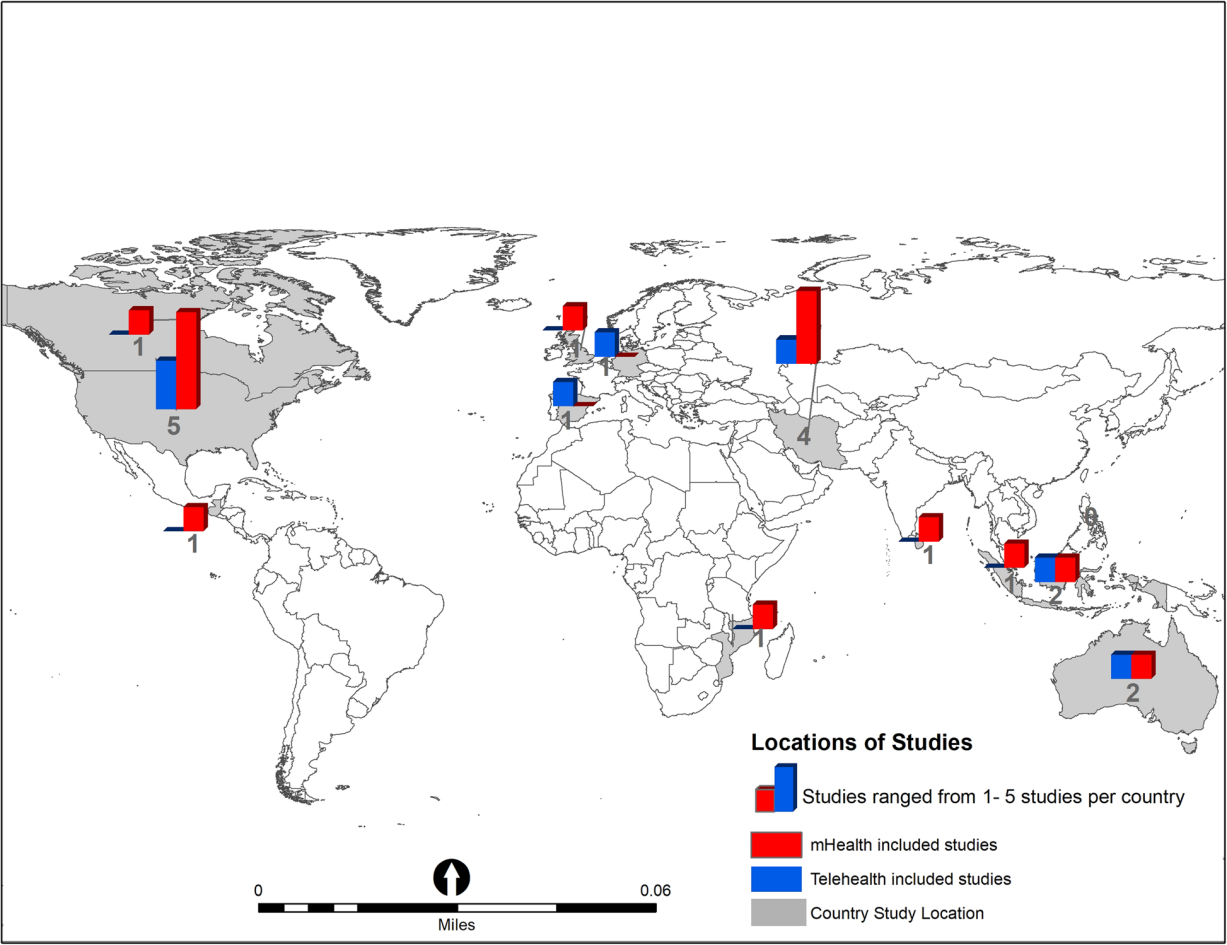
Country loctaion of studies included in the scoping review. Map was genereated using ArcGIS software v. 10.5 (https://www.esri.com/en-us/home)

Table 1 shows the 22 studies that were selected on COVID-19, mHealth and telehealth from the three stages of pregnancy. The included studies were published in English, and between 2020 and 2022. Of the 16 mHealth studies, 7 studies were conducted in the USA (*n* = 4) and Iran (*n* = 3). The remaining 9 studies were conducted in Australia, Canada, Guatemala, Indonesia, Mozambique, Singapore, Sri Lanka and the UK (one study in each country). One study’s location was unclear. Of the 6 telehealth studies, one was conducted in the USA and the rest in Australia, Germany, Indonesia, Iran and Spain (one study in each country). Figure 2 shows the country locations of the peer-reviewed studies included in this scoping review.

**Table 1:** Summary of studies of digital technology-enabled health interventions (mHealth and Telehealth) implemented during the coronavirus crisis to support pregnancy care, globally

Authors	Publication year	Origin	Intervention (s)	Facilitators	Study design	Study population, age, Sample size	Context for facilitators	Outcomes
**mHealth for mental and physical wellness (6 studies)**								
*Pregnancy*								
Kiani & Pirzadeh [96]	2021	Iran	Physical activity educational intervention delivered via mobile app to increase physical activities in pregnant women during the coronavirus crisis.	Perceived app benefits: motivational content and images that emphasize both physical and psychological benefits.	Quasi-experimental study	93 pregnant women aged 16–20 weeks of gestation participating in the childbirth preparation classes.	Training on how to use the application, app did not require an Internet connection.	Mobile Apps increased scores of perceived benefits, barriers, social support, enjoyment, and improved levels of physical activity in pregnant women.
Smith et al. [97]	2021	USA	Consumer-based mobile phone meditation application (app) to help pregnant women self-manage stress and anxiety.	Satisfaction with the Calm app, and easy to use.	Randomized Controlled Trial	Pregnant women 18 years and older with confirmed pregnancy between 14 and 34 weeks of gestation attending a university outpatient clinic of obstetrics and gynecology.	Ability to regularly access a smart device, self-reported use of app; Training on how to download and use the mobile app.	Women who used the prescribed consumer-based mobile meditation app during the coronavirus crisis had significant reductions in perceived stress, depression, anxiety, and sleep disturbance compared with standard care.
Kubo et al. [98]	2021	USA	Self-paced, guided mindfulness meditations provided through a website or mobile application (iOS and Android) Headspace™ app to improve adherence and efficacy in pregnant women.	Convenience of the intervention; ease of use and the short dosage (10–20 min a day).	Single-arm trial	27 pregnant women age ≥ 18 years with moderate-to-moderately severe depression symptoms and < 28 weeks of gestation identified through the electronic health records, and self- or clinician-referral from obstetrics and gynecology clinical staff or study brochures.	Access to a smartphone, tablet, and/or computer with Internet connection.	Improvements observed in pre-postintervention scores for depression symptoms, perceived stress, sleep disturbance, and mindfulness; over half of participants used the app ≥50% of the days during the 6-week intervention.
*Postpartum period*								
Avalos et al. [49]	2020	USA	Self-paced, guided mindfulness meditation training delivered via mobile app Headspace™ app (iOS and Android) or website to deepen mindfulness and encourage routine use in postpartum women.	Convenience of the intervention delivered via commercially available mindfulness app (headspace).	Mixed methods single-arm feasibility trial	13 women aged 18 years; within 6 months of giving birth, with moderate to moderately severe depressive symptom was recruited via electronic health records, self- or clinician referral in obstetrics and gynecology clinics.	Seeking standard postpartum care and access to a smartphone, tablet, or computer with internet access.	Preliminary efficacy and improvement in depression symptoms, perceived stress, sleep quality, and mindfulness in postpartum women with moderate to moderately severe symptoms of PPD.
Dol et al. [99]	2021	Canada	Six-week postpartum mobile phone text message program “Essential Coaching for Every Mother” to improve women’s psychosocial outcomes in the immediate postpartum period.	Acceptability of the program in terms of timing and content; perceived support from the providers.	Prospective pre-post study	88 first time mothers aged 18 years and older between 37 weeks’ gestation or those who gave birth over a three-month period at the study clinic.	Daily access to a mobile phone with text message capabilities.	Essential Coaching for Every Mother mHealth intervention improved maternal self-efficacy and decreasing anxiety: baseline compared to follow-up
Jayasinghe et al. [69]	2022	Sri Lanka	Telephone interviews providing psychological support to pregnant and postpartum women during the pandemic.	Better access to health care; selection bias	Large-scale, population-based pregnancy cohort	1438 (42.6%) pregnant women.	Availability of mobile phone and fixed access to phones; availability of trained interviewees.	476 (33.1%) of interviewed women used messaging apps to receive health messages such as WhatsApp, Viber, IMO, and Facebook Messenger.
**mHealth for health promotion, tracking and education (5 studies)**								
*Pre-pregnancy*								
Leight et al. [100]	2022	Mozambique	Short text message reminders designed to encourage uptake of health facility visits for family planning counselling.	Reminder support; timely clinic visits.	Two arm randomized controlled trial	5623 women of reproductive age who received a referral from a community health worker to visit a health clinic for a family planning consultation.	Availability of a phone number on patient record; PSI-provided smartphone to access the platform.	The effect of the text reminders were positive and statistically significant. Women who received text reminders more likely to visit a clinic, report receiving a contraceptive method at a clinic and prompt visit to a clinic (conditional on ever reporting a visit).
*During Pregnancy*								
Krishnamurti et al. [101]	2021	USA	Prenatal care app based intimate partner violence (IVP) self-screening tool (MyHealthyPregnancy app to encourage continuation of screening and receiving of support services.	Willingness to disclose IPV experiences through an app; social isolation; perceived support.	Quality Improvement Pilot Study	552 pregnant women who used the app during an in-person visit.	Presence of clinical education team; availability of Apple v.1.4.7 and Android v.1.8.	App-based screening captured a distinct set of at risk IPV patients, complementing in-person assessments. Incidence of IPV slightly increased during the shelter-in-place order.
Varnfield et al. [102]	2021	Australia	mHealth platform M♡THer for gestational diabetes mellitus management in pregnancy.	Acceptability of the platform by women; faster interventions.	Evaluation design	23 women with a first-time diagnosis of gestational diabetes mellitus; between 24 and 28 weeks of gestation; at least 16 years old were recruited at a study hospital.	Ownership and ability to use a smart mobile phone	Satisfaction and ease of use of the mHealth platform, with technological challenges around wireless connectivity.
Moulaei et al. [103]	2021	Iran	Mobile-based application developed using Java programming language in an Android Studio programming environment to support self-care and self-management for preeclampsia in pregnancy.	Perceived knowledge and better attitude towards COVID-19 and preeclampsia.	User centered design	10 pregnant women (with/without preeclampsia, and with/without COVID-19 infection) and 10 doctors in affiliate hospitals and medical centers.	Availability of mobile devices with Android operating systems.	Pregnant women rated the usability of the application at a satisfactory level.
Moulaei et al. [104]	2021	Iran	Smart phone-based self-care application developed to help pregnant women against the coronavirus. Educational needs provided via different methods (e.g., texts, educational videos, audios).	Perceived usability; user satisfaction with data elements, educational information needs, functions, and lifestyle information.	Descriptive applied study conducted in two phases	36 pregnant women (> 8 weeks, gestational age 20–50); 11 had a coronavirus diagnosis and without exceptional care, partial or absolute rest recruited at affiliated hospitals.	Daily use of smartphone.	With an average score of 7.94 (out of 9), pregnant women rated the application at a satisfactory level.
**mHealth for parenting support (5 studies)**								
*Pregnancy*								
Ceballos et al. [105]	2020	Guatemala	Text messages (SMS) reminders and phone calls to encourage individuals to visit the health center to monitor the provision of health and nutrition interventions linked to the first 1000 days of life (exclusive breastfeeding, vitamin A, powdered micronutrients, and vaccines).	Availability of health services; absence of social conflicts.	Clustered randomized controlled trial (cRCT)	1542 households with pregnant women and children under two years old who receive key health and nutrition interventions from local public health centers.	Access to mobile phone, presence of at least one child under two years old or one pregnant woman; monthly airtime top-ups.	Response rate to phone calls was 5 times higher compared to text messages (75.8% vs. 14.4%). The cost for mobile phone call reminders were cheaper than that of SMS.
Rhodes et al. [106]	2020	UK	Baby Buddy, a pregnancy and parenting app provides trusted, evidence-based information and self-care tools to help expectant and new parents through pregnancy and the first 24 weeks of parenthood (e.g., being pregnant or parenting a young baby, mood, levels of anxiety, key concerns).	Low literacy level requirements and extensive video content; accessibility to people not in education, training, or employment and those who do not speak English.	Service evaluation study	436 expectant (*n* = 244, 56.0%) and recent (*n* = 192, 44.0%) parents age < 21- older than 45 years who were Baby Buddy app users.	Smartphone ownership: app is free to download and available in all app stores.	97% (423/436) of respondents reported that Baby Buddy was currently helping them. Greater speed in updating digital content to reflect changes due to the pandemic.
Wulandari et al. [107]	2022	Indonesia	Interactive mHealth message intervention via flyers (text, images), videos, and assistance (consultation, discussion, sharing, and question and answer) to improve safe and effective postpartum care.	Perceived easiness to understanding shared information; increased knowledge; existence of communication, interaction, social networks, and the impact of the use of social media.	Quasi-experimental design	46 pairs of pregnant women (gestational age 28–34 weeks) and their husbands were selected purposively from data on pregnancy visits at the Community Health Center.	Availability of mobile phone with WhatsApp application	Knowledge of mothers and husbands increased on post-partum care, and so was improvement in the mother’s practices related to postpartum visits.
*Postpartum period*								
Shorey et al. [108]	2021	Singapore	Supportive Parenting App (SPA, iOS, and Android) development procedure to provide perinatal educational intervention for couples with healthy infants.	Adequate time; financial budgeting and team cohesion.	Multistage iterative development process, and information systems research framework	10 new parents and research team members (app developers, clinicians, and research assistants).	Availability of smartphone with internet access.	Documented the technical details of the SPA and intervention highlights the key aspects needed for future app development.
Quifer-Rada et al. [109]	2022	Not clear	Automated breastfeeding consultation system on LactApp, an mHealth Solution for breastfeeding support	Free mobile app. Self-administered tools; functionalities of breastfeeding monitoring; breastfeeding tests and personalized plans.	Observational, descriptive, and retrospective study	137,327 active users	Availability of email of registered users, demographic factors of mother and baby.	Active users increased by 12, 092; topics of interest for consultations varied but include growth spurts, breastfeeding stages, breastfeeding technique, breast pain and mastitis, problems with infants not gaining weight.
**Telehealth for mental and physical wellness (2 studies)**								
*Pregnancy*								
Hashemzahi et al. [110]	2022	Iran	Self-care training via telemedicine to help mothers familiarize with, manage, and follow up risk symptoms, and to reduce their own stress and anxiety. Audio PowerPoints turned into video content, sent through WhatsApp messenger.		Quasi-experimental study	100 pregnant women aged 18–49 years, with gestational age of 20–28 weeks and referred to comprehensive health centers for pregnancy-related complications and COVID-19 infection.	Having a mobile phone or PC and the ability to use them for Internet access	Findings show that telemedicine COVID-19 self-care training significantly reduced perceived stress, and anxiety in pregnant women including rising awareness about coronavirus and reducing false beliefs.
Silva-Jose et al. [111]	2022	Spain	Virtual supervised exercise program to increase maternal physical activity and improve health outcomes, with classes delivered in an online format using the Zoom platform.	Greater availability of time; home confinement; perceived sense of sense of social support	Evaluation design using semi-structured interviews	24 women between 8 and 10 and 38–39 weeks of pregnancy and attending online fitness classes during the confinement period.	Access to the Zoom platform from home.	Pregnant women were receptive to online group exercise classes and liked the accessible option to accommodating physical activity during the pandemic.
**Telehealth for health promotion, tracking and education (4 studies)**								
*Pregnancy*								
Oelmeier et al. [124]	2022	Germany	Prenatal counseling via Telemedicine: video consultations in a tertiary center for obstetric care that was a part of the larger open Video Service project on telemedicine.	Perceived satisfaction and feasibility.	Prospective single-center trial	75 video consultations were carried out with patients requiring prenatal or pre-pregnancy counseling.	Being a part of a larger open Video Service project on telemedicine.	Patient satisfaction was high (95%, 71/75) but technical problems occurred in 37% (29/75) of the appointments.
Nur et al. [112]	2020	Indonesia	Android-based electronic technology antenatal care (e-ANC) to enhance participation of midwives and pregnant women in antenatal care (e.g., counseling, high-risk early detection on pregnancy, and monitoring of Hb and Fe tablets).	Perceived privacy and confidentiality.	Quasi-experimental study using pre- and post-test experiments	30 pregnant women (in 2nd trimester) ages < 20- > 35, and 20 midwives at areas around the Public Health Centers.	Capacity to use Android devices with the e-ANC feature, speak Indonesian	e-ANC increased prenatal care visits particularly counseling, high-risk early detection, monitoring Hb, and provision of Tablet Fe.
*Postpartum period*								
Palmer et al. [113]	2021	Australia	Telehealth integration into routine antenatal care and delivered via telephone or video conferencing compared to conventionally delivered care on pregnancy outcomes.	Perceived easy of self-monitoring, perceived technology, and communication of appointments support.	An interrupted time-series analysis	2292 women who gave birth between April 20 and July 26, 2020, across a large health service, with large numbers of births assessed in both periods.	Availability of phone or internet with video; implementation of integrated antenatal care.	Telehealth successfully integrated into antenatal care, and it enabled the reduction of in-person consultations by 50% without compromising pregnancy outcomes.
Reisinger-Kindle et al. [114]	2021	USA	Maintenance of telehealth as an option for prenatal and postpartum visits after state-mandated restrictions eased.	Perceive significance in choice of appointment, perceived support from providers regarding use of phone systems, and mandatory virtual meetings.	Retrospective chart review of all pre- and post-natal care visits	558 prenatal patients and 209 postpartum patients receiving prenatal or postpartum care at a large urban academic obstetrics and gynecology practice.	Availability of video equipment for those with video telehealth capabilities, and/or availability of audio-only telephone; primary language Spanish; Availability of trained providers.	The Reach of the intervention increased from baseline. Adoption was high, with all thirty providers using telehealth, and the telehealth found to be feasible and acceptable based on uptake. Effectiveness measures suggest potential for earlier diagnosis of prenatal conditions.

The 16 mHealth included studies were classified into four types of study designs: experimental studies (11 studies, including 5 randomized control trials (RCTs), and 2 quasi-experimental studies), one was a mixed methods study, and two were observational studies. Two of 16 mHealth studies used a multistage iterative development process and a user centered design. We then grouped the identified 6 telehealth interventions into three types of study designs: experinmental studies (2 studies), evalaution studies (3 studies), and one study was a retrospective chart review of all pre- and post-natal care visits. All included studies reported the use of mHealth or telehealth to support the three stages of pregancy during the COVID-19 pandemic.

Although included studies (telehealth and mHealth) reported varying degrees of eligiblity criteria for selecting study participants, women had to be pregnant or should have given birth within a stipulated study period. Study particpants in most studies were selected based on the pregnant woman’s age (e.g., at least 18 years), their gestational age in weeks or months (e.g., 16–20 weeks of gestation), or if the pregnant woman was a parent with a child born within 6 months postpartum. Two articles included women of reproductive age. A few studies selected pregnant women based on their area of residence and/or service clinic or if women were active users of the study digital technolgy.

### Overview of interventions examined

**Fig. 3:**
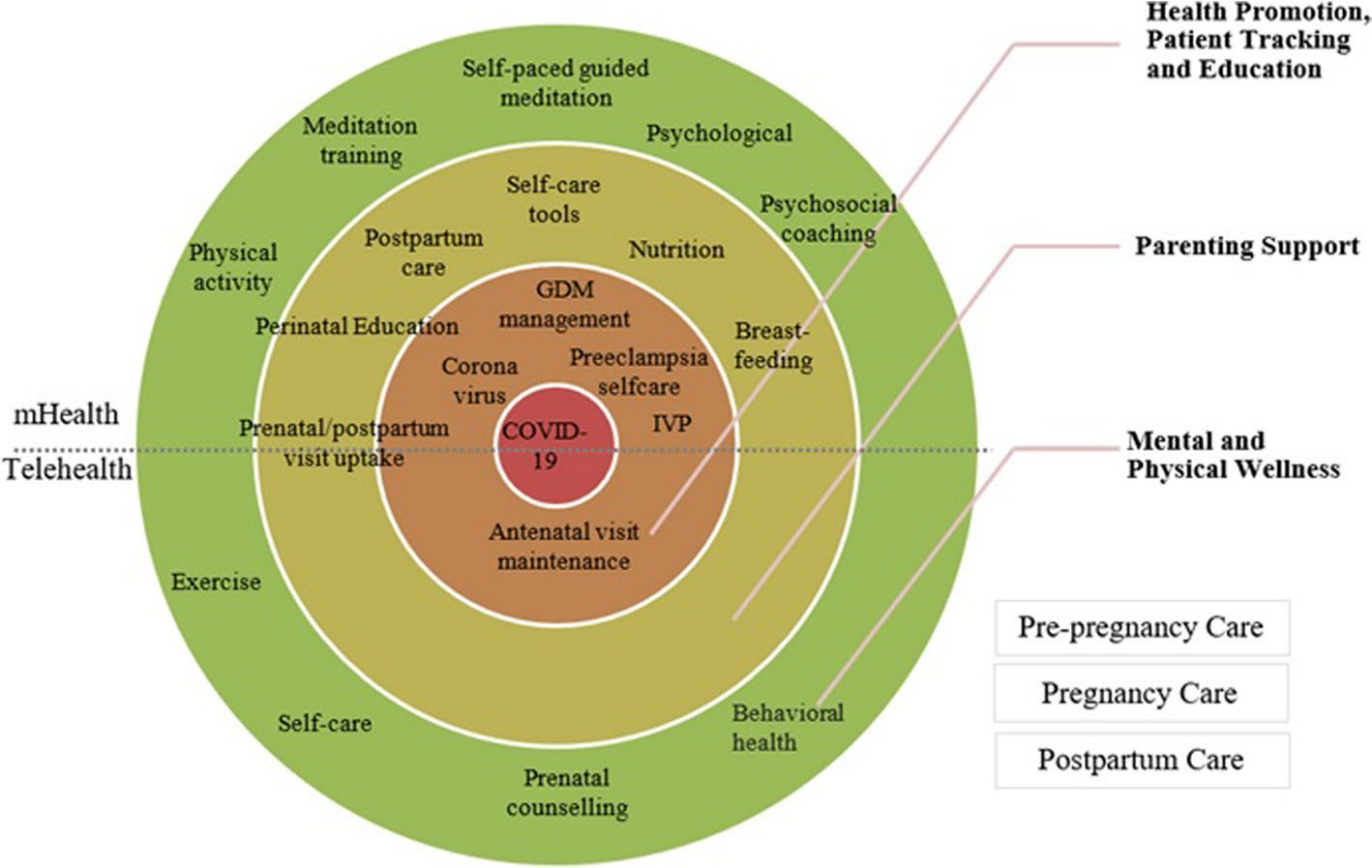
Digital interventions (mHealth and telehealth) implemented at different stages of pregancy in the inlcuded studies

To classify the chosen studies, we applied the program theory of mHealth programs and maternal and child health [43] and developed a modified model of the major outcomes (Fig. 2). The included 22 studies reported on three outcomes: 1) mental and physical wellness, 2) health promotion, patient tracking and education, and 3) parenting support (Fig. 3). The mHealth interventions were delivered using various technology platforms, including mobile app (iOS and Android) or smartphone (15 studies) with some sending intervention content via WhatsApp Messenger and/or email (1 study). A few studies used a combination of mobile phone or smart phone and/or websites.

### Mental and physical wellness

A recent review of the psychological impact of COVID-19 pandemic on the mental health of pregnant women, conducted by researchers at the University of Cagliari in Italy in 2021, linked an increase in mental health of pregnant women to the coronavirus pandemic [115] and underscored the need for psychological support during pregnancy to mitigate mental health and the risk of long-term impacts on child development. Three of six mHealth mental and physical wellness studies supported pregnant women (3 studies) while three studies supported postpartum care. Mental and physical wellness interventions included: 1) self-paced guided mediation, 2) meditation training, 3) psychological support, 4) psychosocial coaching, and 5) physical activity.

#### mHealth mental and physical wellness interventions for pregnancy support

To determine the effect of a mobile application (app)-based health interventions, with motivational multimedia with photos and videos on physical activity, Kiani and Pirzadeh [96] conducted a quasi-experimental study of 93 pregnant women (16–20 weeks of gestation) participating in childbirth preparation classes during the coronavirus crisis. Researchers encouraged women to use the mobile app designed not to require internet connection for a specified period. Findings (pre- and 3 months-post intervention) showed that the perceived benefits and enjoyment of physical activity increased post intervention in the intervention group (compared to the control group), so did the mean score of physical activity in this group. Smith and colleagues evaluated the effect of a consumer-based mobile app-based meditation on wellness by randomizing 101 women (50 women in the treatment group, and 51 women in the control group and standard care). Pregnant women in the treatment group used the mindfulness meditation app Calm for 30 days. Results showed a significant reduction in the intervention group (compared to the control group) in perceived stress, depression, anxiety, and sleep disturbance [97].

#### mHealth mental and physical wellness interventions for postpartum support

To evaluate the effect of a six-week postpartum mobile phone text message program (“Essential Coaching for Every Mother”) on maternal self-efficacy, social support, postpartum anxiety, and depression, Dol and colleagues [99] conducted a prospective study with 88 first-time mothers enrolled after giving birth and 6 weeks postpartum between July 15 and September 19, 2020. The study noted an increase in self-efficacy (at follow-up compared to baseline), and a reduction in anxiety, and women’s satisfaction with the program [99]. However, further work is required to establish the viability of this program. Using a large-scale, population-based pregnancy cohort, Jayasinghe and colleagues [68] recruited 3374 first-trimester pregnant women registered with midwives at the field prenatal clinics to assess the feasibility and the coverage and feasibility of app-based interventions and generalizability of telephone interviews for psychological support during the pandemic. The study revealed that mHealth led to selection bias and that mHealth may not be the best strategy for interventions in this remote area.

Two studies in the US tested the feasibility and acceptability of offering a self-paced, commercially available mobile-app (Headspace™)-based mindfulness intervention in women with depressive symptoms by using single arm trials [48, 98]. Notably, although one of these studies focused on pregnant women (< 28 weeks of gestation who were not practicing a regular, < 3 times per week) mindfulness/meditation [98]; the other study focused on postpartum women [48]. Both studies noted significant improvements in pre-post-intervention scores for depression symptoms, perceived stress, sleep disturbance, mindfulness, feasibility, and acceptability of the mHealth mindfulness intervention for pregnant and postpartum women. In both studies, women had to follow a self-paced, 6-week mindfulness meditation program using the app 10–20 minutes each day over the 6-week period [98].

### Health promotion, tracking and patient education

Health education during pregnancy plays a critical role in improving maternal and neonatal outcomes (e.g., birth weight, initiation and continuation of breastfeeding, and postpartum strategies) [116–119]. Five of the 16 mHealth studies focused on health promotion, tracking and patient education to support the three stages of pregnancy [100]. These interventions focused on educating women regarding the coronavirus, management of gestational diabetes, preeclampsia, perinatal education, visits, and maintenance including uptake.

#### mHealth for health promotion, tracking for pre- pregnancy support

To estimate the effects of mobile text messages in encouraging the use of family planning services in Mozambique, Leight and colleagues [100] conducted a randomized controlled trial with 5623 women receiving services from the Integrated Family Planning Program implemented by Population Services International between 20 January and 18 December 2020. Women in the treatment group received a series of text message reminders encouraging uptake of health facility visits for family planning counselling. They observed that women in the treatment group were more likely to receive a contraceptive method at a clinic.

### mHealth for health promotion, tracking and pregnancy support

A quality improvement study that screened 552 patients for intimate partner violence (IPV) during the COVID-19 pandemic using a prenatal care app which showed that the use of the IPV screening tool increased during the lock-down period [101]. Another evaluation study focused on the adoption and multidisciplinary care coordination of an mHealth platform in a cohort of women with first-time diagnosis of GDM showed that high blood glucose reviews and antenatal contact among app users (compared to non-app users) [102].

Two studies conducted in Iran designed and developed two mobile apps to facilitate self-care for pregnant women with preeclampsia during COVID-19, and examined the effect of a self-care smartphone-based application on self-care practices for pregnant women against COVID-19. Both studies noted positive reviews, and the apps were rated highly by users [103, 104].

### Parenting support

Parenting can be tough, and parenting apps can lessen the burden for first-time parents through providing access to information and tracking child development [120] especially during the COVID-19 pandemic, when pregnant parents and parents of young children lost usual support networks. Five studies focused on parenting support during pregnancy (3 studies) and the postpartum period (2 studies), and four interventions enabled by mHealth technology: 1) breastfeeding, 2) nutrition support, 3) self-care tools, 4) postpartum care, perinatal education and/or uptake.

#### mHealth for parenting support

Ceballos and colleagues [105] applied a clustered randomized controlled trial (cRCT) to evaluate the feasibility of using simple calendarized mobile text messages (SMS) and phone calls to improve the timely reception of health services of key health and nutrition interventions linked to a critical period of growth and development (the first 1000 days of life), but did not find any effects. However, mobile phone calls were an effective low-cost tool. Rhodes and colleagues [106] conducted a service evaluation using mixed methods to determine whether the “Baby Buddy”, a pregnancy and parenting app aimed at supporting pregnant women and new parents through the first 24 weeks of parenthood, was meeting users’ needs (436 women). Women reported increased anxiety due to decreased health care delivery and loss of social support from friends and family. In Indonesia, a quasi-experimental study assessed the effectiveness of an interactive mHealth message intervention (text, images), videos, and assistance (consultation, discussion, sharing, and question and answer) using WhatsApp groups to improve postpartum care behavior of 88 mothers and husbands (43 pairs- treatment group and 45 pairs - control group) [107]. The duration of the intervention was 14 days (5 hours per day, followed by random flyers delivered at birth up to 42 days post birth). The researchers report improved postpartum care behavior for mothers and their husbands.

#### mHealth for parenting support during the postpartum period

One study developed a Supportive Parenting App (SPA) to improve parent and infant outcomes in the perinatal period, and highlight the challenges and lessons learned, which centered around user and technological problems [108]. Likewise, a study in an unspecified location evaluated whether the coronavirus crisis affected breastfeeding consultations by using data from LactApp (a mobile application [app], an mHealth solution designed for breastfeeding support, and revealed an increase in breastfeeding support [109].

### Telehealth for mental and physical wellness

Previous studies conducted prior to the pandemic as well as during the pandemic has demonstrated the significance of telemedicine prenatal care [121, 122]. However, others have raised concerns regarding equity in access to care, particularly in resource-limited settings [123]. The 6-telehealth technology-enable interventions identified in this scoping review focused on exercise, selfcare, prenatal counseling and behavioral health.

#### Telehealth for mental and physical wellness support during pregnancy

To explore the experiences of pregnant women (8–39 weeks of pregnancy) participating in an online group exercise program, and the role of a virtual group fitness on maternal mental outcomes, Silva-Jose and colleagues [111] applied a phenomenological approach in study conducted between March to October 2020. The researchers found that pregnant women were receptive to the online group exercise classes and liked the accessibility of the physical activity, and the social connection they provided. Three of the telehealth studies focused on antenatal care. One study quasi-experimental study with 100 pregnant women aged 18–49 years and 20–28 weeks gestation age determined the effect of COVID-19 self-care training via telemedicine on perceived stress and corona disease anxiety, and noted a reduction in perceived stress in the treatment group (compared to the control group) [110]. Findings confirm the effectiveness of the selfcare training implemented via telemedicine and during the coronavirus crisis in reducing the perceived stress and anxiety of pregnant women.

#### Telehealth for health promotion, tracking and education support during pregnancy

Regarding the telehealth technology-enable interventions, Oelmeier and colleagues [124] conducted a prospective single-center trial by use of 75 video consultations in a tertiary center for obstetric care, and the results indicated that patient satisfaction was high, but technical problems were experienced in 37% of the appointments. Nur and colleagues [112] used a purposive sampling technique and post-test experiments on 30 pregnant women and 20 midwives to examine the effect of COVID-19 on antenatal visits on pregnant women. They found differences in the prenatal visits among pregnant women pre-and post the COVID-19 lockdown period, and in midwives participation rates in counseling, high-risk early detection on pregnancy, Hb monitoring, and provision of Fe tablets.

#### Telehealth for health promotion, tracking and education support during the postpartum period

In Australia, one telehealth technology study used an interrupted time-series design to assess the impact of telehealth integration into antenatal care by comparing the first 3 months of telehealth integrated care delivered between April 20 and July 26, 2020, with conventional care across low-risk and high-risk care models. We found no significant differences in the integrated care period as it relates to the number of babies with fetal growth restriction (e.g., birthweight) or pregnancies complicated by pre-eclampsia, or gestational diabetes. Overall, they noted a reduction in-person interactions during the pandemic, and recommend the use of telehealth in post-pandemic health-care models [113]. Further, a retrospective chart review of electronic health records of all pre-and postnatal care visit encounters (558 patients, and 1788 prenatal visits) from March 19 and August 31, 2020 noted limited effectiveness measures but potential for earlier diagnosis of some prenatal conditions, and that telehealth was a feasible option [114].

## Discussion

We performed a global scoping review to synthesize the published peer-reviewed literature on the mHealth and telehealth studies conducted during the coronavirus crisis to support pregnancy care. These studies showed how mHealth and telehealth studies varied regarding the types of designs, context, interventions and/or modalities, uptake facilitators and outcomes studied.

mHealth studies constituted 72.7% of the published studies while 27.3% were telehealth studies. There were only 3 example studies that integrated digital technologies into healthcare systems [28, 98, 113] and only 3 studies that designed, developed and evaluated the feasibility of mobile apps to support one or a combination of the 3 stages of pregnancy [103, 104, 108]. Mobile apps and short-messaging services were the primary technologies employed in mHealth studies. Experimental studies, such as (RCTs) quasi-experimental studies accounted for 68.8% of mHealth studies, and only 33.3% were telehealth studies using experimental study designs. Future studies that determine whether a cause-effect relation exists between treatment and outcome during pandemics or emergencies are therefore recommended.

We identified three key functionalities of pregnancy apps and telehealth platforms including mental and physical wellness, health promotion, patient tracking, health education, and parenting support. However, the results of the synthesized studies demonstrate the similarities and differences between participant characteristics and intervention modalities used from prenatal through delivery and postpartum. For instance, there were considerable differences in study participants ages, with most studies using both the birth age of the mother and gestation age, while a few studies did not include age but referenced the age of participants as women of reproductive age [100] or pregnant women [68, 101, 103, 109, 124, 125], or women with children under 2 years old [105, 113, 126]. Likewise, although two studies conducted in the US used a similar mHealth intervention delivered via Headspace™ app and with patients with moderate-to-moderately-severe depression as well as duration (use of app for 10 to 20 minutes a day for 6 weeks), they focused on different stages of pregnancy, gestation age and patient birth ages. For example, Avalos and colleagues [48] applied the mHealth mindfulness program to support women aged at least 18 years, and within 6 months of giving birth, while Kubo and colleagues [98] applied it to support pregnant women age 18 years or older with less than 28 weeks of gestation.

In this scoping review, 37.5% of included studies utilized mHealth for physical activity education, mental health and wellness, mindfulness meditation, psychological and psychosocial (e.g., maternal self-efficacy, social support, anxiety -postpartum specific and coronavirus related stress). This provides noble interest and motivation of using mHealth technologies and content-driven education to facilitate maternal self-efficacy. In addition, although only few studies were included, the finding highlights the psychological sequelae of COVID-19 on pregnant women, a concern raised in recent studies [19]. The identified mHealth and telehealth platforms from this scoping review provide good evidence as digital technologies used by both women and providers to support maternity continuum of care during the pandemic. Similarly, the design of self-paced guided and instances of supervised self-care, including the use of apps without needing internet connection can enhance uptake, promote healthy pregnancies and replication in other subpopulations and clinical settings. Our findings are in line with pregnancy apps desired functionalities reported in other related reviews [80, 83, 127]. Moreover, interventions were piloted and evaluated with potential users (women and providers) before implementation.

Previous studies have indicated the enormous effect that positive parenting practices can have on children’s social, emotional, and intellectual development, especially during the early years, and parenting apps can help first-time parents access information and track child development. Five of the 16 mHealth studies focused on this important topic and revealed that mHealth technologies play a key role in supporting new parents to access information and receive health interventions because of their ease of accessibility. However, only two of the five mHealth parenting studies used experimental study designs, therefore questions remain.

In terms of the telehealth studies, pregnancy, and the COVID-19 pandemic, 37.5% included studies supported pregnancy and postpartum care via prenatal counselling, self-care training, supervised exercises, prenatal care provision, and or maintenance and behavioral health. Most studies used a combination of platforms in the delivery of telehealth interventions such as video consultations, audio PowerPoints turned into video content and sent via mobile WhatsApp, Zoom platform, or telephone. What is surprising is the limited evidence supporting telehealth interventions in obstetrics and gynecology, a finding also reported by a systematic review conducted before the COVID-19 pandemic [60].

In summary, although our scoping review has limitations that are embedded from the included studies to our knowledge, the current study is the first to uncover context, digital interventions, uptake facilitators, locations, study designs and outcomes in the interventions implemented during the COVID-19 pandemic to support pregnancy care. We recognized three key functionalities of both pregnancy apps and telehealth that have been studied to date, including clinical outcomes across the maternity care continuum that has been supported using the two digital technologies during the coronavirus crisis to support pregnancy care based on the theory for mHealth programs.

## Conclusion

Our scoping review identified key functionalities of mobile apps and telehealth platforms, pregnancy and intervention outcomes, context, interventions, study designs and approaches in the included studies during the COVID-19 crisis. Most studies focused on mental health and physical wellness, and mobile phone apps were the most used modality, followed by telehealth. Few studies included the use of WhatApp, messenger and websites. Regarding methodological approaches, slightly more than half used experimental studies, and the remaining were evaluation studies, developmental or design studies. Included studies published in the past two and a half years underscore an emergent topic of study, and we anticipate more studies in the near future.

## Supplementary Information

**Additional file 1: Appendix A.** Keyword search strategy.

## Data Availability

All sharable data have been uploaded.

## Abbreviations

apps: Applications
COVID-19: Coronavirus Disease 2019
cRCT: Clustered randomized controlled trial
Fe: Iron
GDM: Gestational Diabetes Mellitus
Hb: Heartbeat
iOS: IPhone operating system
IPV: Intimate partner violence
mobile app: Mobile application
mHealth: Mobile health
PRISMA: Preferred Reporting Items for Systematic reviews and Meta-Analyses extension for Scoping Reviews
RCT: Randomized controlled trial
SDGs: United Nations Sustainable Development Goals
SMS: Short message service / text message
SPA: Supportive Parenting Application
UK: United Kingdom
US: United States
USA: United States of America
