# Supplementary material for: Lessons from digital technology-enabled health interventions implemented during the coronavirus pandemic to improve maternal and birth outcomes: a global scoping review
Source: BMC Pregnancy Childbirth. 2023 Mar 20;23:195. doi: 10.1186/s12884-023-05454-3 (PMC10026210; doi:10.1186/s12884-023-05454-3)
Supplement: Supplementary file 1 — Additional file 1: Appendix A. Keyword search strategy. [file 12884_2023_5454_MOESM1_ESM.docx]

## APPENDIX A: KEYWORD SEARCH STRATEGY

| **Concepts** | **(P) Study Population** | **(I) mHealth Intervention** |
| --- | --- | --- |
| Author Keywords | Maternity  pregnan*  prenatal  antenatal  obstetric  postpartum  new born  follow up | mHealth  “m health”  eHealth  “e Health”  Telehealth  “mobile health”  Mobile app*  Mobile technology  Mobile phone  Smartphone*  Cellular phone*  Cell phone*  Tablet*  Personal digital assistant  PDA  Wearable  Biosensor*  Portable electronic application*  Portable software application*  Text messages  Phone app*  Cellphone app*  Telephone app*  Remote monitoring  Remote patient monitoring  Remote patient management  Remote biometric monitoring  Telenephro*  Tele-nephro*  Telecare  Telecommunication* |
| Subject Headings: MeSH | Education  Vaccination  Screening  Assistance  pregnancy tests  services | Telemedicine[MeSH]  Smartphone[MeSH]  Mobile applications[MeSH] |

**MEDLINE/PubMed Search Statement**

Maternity OR pregnan* OR prenatal OR antenatal OR obstetric OR postpartum OR newborn OR neonatal care **AND** Intervention OR pregnancy tests OR prenatal care follow up OR pregnancy follow up OR prenatal education OR vaccination OR screening OR child assistance **AND** COVID-19 OR 2019 novel coronavirus OR Wuhan coronavirus OR novel coronavirus OR Wuhan virus **AND** mHealth.mp OR mobile application.mp OR Exp Mobile Applications OR smart phone.mp OR Exp Smartphone OR Decision aid$.mp OR Risk assessment tool$.mp OR Predictive model.mp OR App.mp OR text messages OR phone app* OR cellphone app* OR telephone app* OR remote monitoring OR remote patient monitoring OR remote patient management OR telecare OR telecommunication* OR telemedicine[MeSH] OR smartphone[MeSH] OR mobile applications[MeSH])

**ProQuest Search Statement**

TS(“Maternity” OR pregnan* OR “prenatal” OR “antenatal” OR “obstetric” OR “postpartum” OR “newborn” OR “neonatal care”) **AND** TS(intervention* OR “pregnancy tests” OR “prenatal care follow up” OR “pregnancy follow up” OR “prenatal education” OR “vaccination” OR “screening” OR “child assistance”) **AND** TS(COVID-19* OR “2019 novel coronavirus” OR “Wuhan coronavirus” OR “novel coronavirus” OR “Wuhan virus”) **AND** TS(mobile phone* OR “mHealth.mp” OR “mobile application.mp” OR “Exp Mobile Applications” OR “smart phone.mp” OR “Exp Smartphone” OR “Decision aid$.mp” OR “Risk assessment tool$.mp” OR “predictive model.mp” OR “App.mp” OR text messages* OR “phone app*” OR cellphone app* OR telephone app* OR “remote monitoring” OR “remote patient monitoring” OR “remote patient management” OR “telecare” OR telecommunication*)

**Web of Science Search Statement**

TS=(“Maternity” OR pregnan* OR “prenatal” OR “antenatal” OR “obstetric” OR “postpartum” OR “newborn” OR “neonatal care”) **AND** TS=(intervention* OR “pregnancy tests” OR “prenatal care follow up” OR “pregnancy follow up” OR “prenatal education” OR “vaccination” OR “screening” OR “child assistance”) **AND** TS=(COVID-19* OR “2019 novel coronavirus” OR “Wuhan coronavirus” OR “novel coronavirus” OR “Wuhan virus”) **AND** TS=(mobile phone* OR “mHealth.mp” OR “mobile application.mp” OR “Exp Mobile Applications” OR “smart phone.mp” OR “Exp Smartphone” OR “Decision aid$.mp” OR “Risk assessment tool$.mp” OR “predictive model.mp” OR “App.mp” OR text messages* OR “phone app*” OR cellphone app* OR telephone app* OR “remote monitoring” OR “remote patient monitoring” OR “remote patient management” OR “telecare” OR telecommunication*).
